# Supplementary material for: Multiplexing Quadrupole and Ion Trap Operation Modes on a “Brick” Miniature Mass Spectrometer
Source: Molecules. 2023 Nov 17;28(22):7640. doi: 10.3390/molecules28227640 (PMC10674918; doi:10.3390/molecules28227640)

Supporting Information

Multiplexing Quadrupole and Ion Trap Operation Modes on a "Brick" Miniature Mass Spectrometer

Chaohong Feng[1], Siyu Liu[1], Ting Jiang[1*], Wei Xu[1*]

[1]School of Medical Technology, Beijing Institute of Technology, Beijing 100081, China

*Corresponding Authors:

Ting Jiang	Wei Xu
School of Medical Technology	School of Medical Technology
Beijing Institute of Technology	Beijing Institute of Technology
Haidian, Beijing, 100081, China	Haidian, Beijing, 100081, China
Email: 6120230051@bit.edu.cn	Email: weixu@bit.edu.cn

Table S1 The components of high-order fields in QMF-1 to QMF-8

Table S1. High-order fields of different QMFs

QMF	A2	A3	A4	A5	A6	A8	A10	A12
QMF-1	1	$-9.83*10^{-7}$	0.0115	$-1.19*10^{-6}$	0.0253	0.0073	-0.0334	-0.0207
QMF-2	0.9813	$-8.41*10^{-7}$	0.0122	$3.74*10^{-8}$	0.0507	0.0181	-0.0310	-0.0118
QMF-3	0.9833	$-2.47*10^{-6}$	0.0106	$6.13*10^{-6}$	0.0172	0.0044	-0.0167	-0.0334
QMF-4	0.9806	$2.68*10^{-6}$	0.0226	$-3.42*10^{-7}$	0.0228	0.0065	-0.0155	-0.0333
QMF-5	0.9823	$1.66*10^{-6}$	0.0131	$2.46*10^{-6}$	0.0272	0.0135	-0.0232	-0.0269
QMF-6	0.9801	$1.93*10^{-6}$	0.0225	$2.99*10^{-6}$	0.0265	0.0095	-0.0176	-0.0312
QMF-7	0.9827	$-2.87*10^{-6}$	0.0111	$-2.44*10^{-6}$	0.0212	0.0075	-0.0189	-0.0314
**QMF-8**	**0.9742**	**$-1.45*10^{-6}$**	**0.0156**	**$-7.90*10^{-7}$**	**0.0410**	**0.0232**	**-0.0257**	**-0.0191**

Table S2 the MS resolution and ion transmission ratios of QMF-1 to QMF-8

Table S2. Different QMFs

QMF	w1(mm)	w2(mm)	d1(mm)	r_x(mm)	r_y(mm)	resolution	Transmission[%]
QMF-1	0.5	3	1.5	5	4.1	176	15
QMF-2	0.5	2.5	1.5	5.5	4.6	77	27.4
QMF-3	0.5	2.5	1	5	3.6	102	18.6
QMF-4	0.5	2.5	1	5	3.7	240	7
QMF-5	0.5	2.6	0.9	5.2	3.9	180	15
QMF-6	0.5	2.6	1	5.2	3.9	180	7.6
QMF-7	0.5	2.6	1	5.2	3.8	120	21.4
**QMF-8**	**0.5**	**2.5**	**0.8**	**5.2**	**4**	**145**	**20**

Figure S1 Influence of guide electrodes on signal intensity

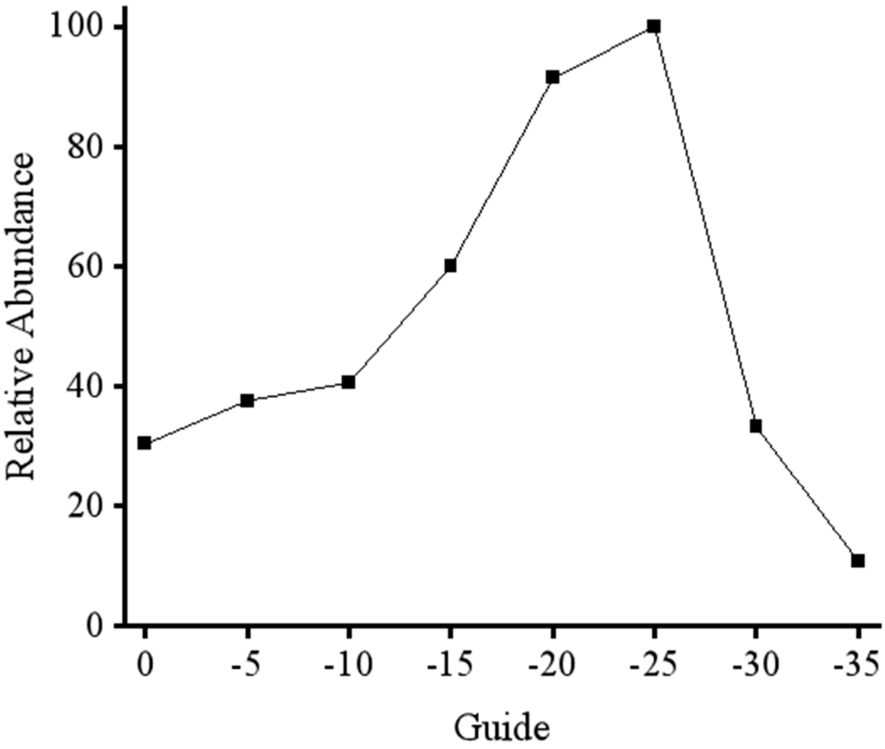

Figure S2 The 3D assembly of the whole instrument.

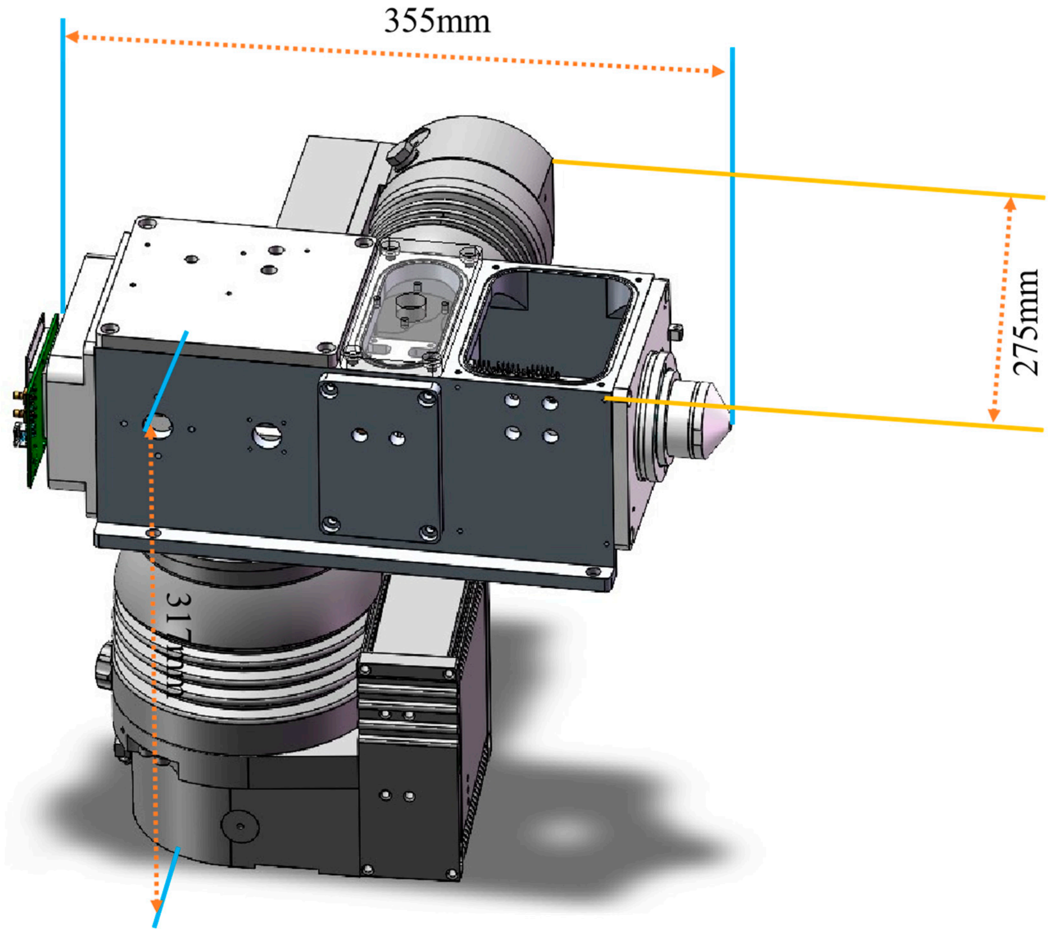

Supplement: Supplementary file 1 [file molecules-28-07640-s001.zip › molecules-2650411-supplementary.pdf]
